# microRNA-141 inhibits cell proliferation and invasion and promotes apoptosis by targeting hepatocyte nuclear factor-3β in hepatocellular carcinoma cells

**DOI:** 10.1186/1471-2407-14-879

**Published:** 2014-11-25

**Authors:** Li Lin, Hongwei Liang, Yanbo Wang, Xiaomao Yin, Yanwei Hu, Jinlan Huang, Tingyu Ren, Hui Xu, Lei Zheng, Xi Chen

**Affiliations:** Department of Laboratory Medicine, Nanfang Hospital, Southern Medical University, North of Guangzhou avenue No.1838, Baiyun District, Guangzhou, 510515 P.R. China; Jiangsu Engineering Research Center for microRNA Biology and Biotechnology, State Key Laboratory of Pharmaceutical, Biotechnology, School of Life Sciences, Nanjing University, 22 Hankou Road, Nanjing, 210093 P.R. China; Qingyuan Traditional Chinese Medicine Hospital, No.11 of Qiaobei avenue, Qiangyuan, 511518 P.R. China

**Keywords:** HNF-3β, miR-141, HCC, Proliferation, Invasion, Apoptosis

## Abstract

**Background:**

Hepatocyte nuclear factor-3β (HNF-3β) plays a critical role in hepatocyte differentiation and controls liver-specific gene expression during the development of hepatocellular carcinoma (HCC), but the molecular basis of this process has not been fully elucidated. microRNAs (miRNAs) are powerful, post-transcriptional regulators of gene expression. Whether miRNAs can impact the effects of HNF-3β in HCC is still unknown.

**Methods:**

HNF-3β and miR-141 expression levels were detected in HepG2 cells, using real-time quantitative RT-PCR (qRT-PCR). Luciferase reporter assays and Western blots were used to validate HNF-3β as a direct target gene of miR-141. Cell proliferation, invasion, and apoptosis were also examined to confirm whether miR-141 could impact on HNF-3β in HCC.

**Results:**

In this study, we found that HNF-3β protein levels were consistently upregulated in HCC clinical tissues compared with matched normal adjacent tissues. However, the mRNA levels of HNF-3β varied in random tissues, suggesting that a post-transcriptional mechanism was involved in its regulation. We used bioinformatic analyses to search for miRNAs that could potentially target HNF-3β, and identified specific targeting sites for miR-141 in the 3′-untranslated region (3′-UTR) of the HNF-3β gene. By overexpressing miR-141 in HepG2 cells, we experimentally validated that miR-141 directly regulated HNF-3β expression. Furthermore, the biological consequences of targeting HNF-3β by miR-141 were examined using cell proliferation, invasion and apoptosis assays *in vitro*. We demonstrated that the repression of HNF-3β by miR-141 suppressed the proliferation and invasion and promoted the apoptosis of HepG2 cells.

**Conclusions:**

miR-141 functions as a tumor suppressor in HCC cells through the inhibition of HNF-3β translation.

## Background

Hepatocellular carcinoma (HCC) is one of the most lethal malignancies and is the third-most common cause of cancer-related mortality in the world [1]. Early-stage HCC with preserved liver function can be effectively treated by resection, liver transplantation or percutaneously and with a more ideal 5-year survival rate [2]. Generally, HCC progression can be defined by a decrease in differentiation, the loss of tissue-specific gene expression, acceleration of cell proliferation and, ultimately, metastasis [3]. Patients with HCC often exhibit tumor cell invasion and metastasis before conventional diagnosis [4]. Therefore, it is vital to study the molecular basis of HCC and explore new therapeutic agents.

The maintenance of hepatocyte differentiation and control of liver-specific gene expression is attributed, in large part, to hepatocyte nuclear factor 3 (HNF-3). The HNF-3/forkhead family of transcription factors in mammals include three genes designated as *HNF-3α* (*Foxa-1*), *HNF-3β* (*Foxa-2*) and *HNF-3γ* (*Foxa-3*), which share homology in their winged-helix DNA binding domains [5]. The HNF-3β gene is located in chromosome 20p11.21, and the downregulation of HNF-3β is associated with apoptotic injury. The overexpression of HNF-3β decreases apoptosis, whereas siRNA silencing of HNF-3β increases apoptosis of HepG2 cells [6, 7]. Recently, some studies have shown that HNF-3β expression and activity are regulated at the post-transcriptional level [8, 9]. For example, Baroukh et al. found that miR-124a can regulate the HNF-3β protein level, but not the HNF-3β mRNA level in pancreatic beta-cell lines [8]. However, the mechanisms of HNF-3β, as well as the clinical and prognostic significance of HNF-3β expression, have never been thoroughly studied in HCC.

miRNAs are non-coding, small, endogenous RNAs approximately 22 nucleotide long that regulate target gene expression at the post-transcriptional level [10–12]. Mature miRNA may inhibit translation of the targeted mRNAs or induce their degradation by preferentially interacting with the 3′-untranslated regions (3′-UTRs) of target mRNAs [13, 14]. Recent studies have demonstrated that abnormal miRNA expression plays an important role in the formation of a wide variety of tumors and is directly involved in the occurrence, development, diagnosis and staging of HCC [15–17]. Fan et al. [18] found that miR-122 was downregulated in the HBV-related HCC cell line HepG2.2.15 and played an important role in HBV-related hepatocarcinogenesis by targeting DNRG3. Li et al. [19] found that miR-429 was upregulated in HCC and that the epigenetic modification of miR-429 could manipulate liver tumor-initiating cells by targeting the RBBP4/E2F1/OCT4 axis. Zhao et al. [20] found that miR-26b suppressed NF-kappa B signaling and, thereby, sensitized HCC cells to doxorubicin-induced apoptosis by the expression of TAK1 and TAB3.

Although HNF-3β and miRNAs are associated with HCC carcinogenesis, little is known about the natural miRNAs that act on HNF-3β. In this study, we found that HNF-3β was directly regulated by miR-141 in HCC cells. Furthermore, we showed that miR-141 inhibited HNF-3β expression to suppress the proliferation and invasion and promote the apoptosis of HCC cells.

## Methods

### HCC specimens

Twelve HCC patients who underwent primary surgical resection were enrolled in this study. Paired HCC and adjacent non-tumor tissue specimens were obtained from consenting patients and were approved by the Medical Ethics Committee of the Southern Medical University. None of the patients had received radiotherapy or chemotherapy before surgery. Clinical and pathological data, including pathological grading and HBV infection are listed in Table 1. Tissue fragments were immediately frozen in liquid nitrogen at the time of surgery and stored at −80°C.

**Table 1:** **Clinical features of hepatocellular carcinoma patients**

	Tumor subtype	Pathological stage	HBV infection
Case #1	HCC	II	HBV+(004)
Case #2	HCC	II	HBV+(005)
Case #3	HCC	III	HBV+(93.57)
Case #4	HCC	II	HBV+(>225)
Case #5	HCC	I ~ II	HBV+(31.83)
Case #6	HCC	II	HBV+(>225)
Case #7	HCC	I ~ II	HBV+(25.723)
Case #8	HCC	I	HBV+(>225)
Case #9	HCC	II ~ III	HBV+(9.102)
Case #10	HCC	III	HBV+(11.687)
Case #11	HCC	II	—(0.001)
Case #12	HCC	II	HBV+(72.519)

### Cell culture

The human HCC cell line HepG2 and Huh7 were purchased from the Shanghai Institute of Biochemistry and Cell Biology of the Chinese Academy of Sciences (Shanghai, China). The cells were cultured in Dulbecco’s Modified Eagle’s Medium (DMEM; Gibco, CA, USA) supplemented with 10% fetal bovine serum (FBS; Gibco) and 1% Penicillin-Streptomycin (Gibco) within a humidified atmosphere containing 5% CO_2_ at 37°C.

### RNA isolation and quantitative RT-PCR

Total RNA was extracted from the cultured cells and human tissues using TRIzol Reagent (Invitrogen, Carlsbad, CA) according to the manufacturer’s instructions. Assays to quantify miRNAs were performed using TaqMan miRNA probes (Applied Biosystems, Foster City, CA) according to the manufacturer’s instructions, and RT-PCR reactions were carried out using the manufacturer’s recommendation. Briefly, 1 μg of total RNA was reverse-transcribed to cDNA using AMV reverse transcriptase (TaKaRa, Dalian, China) and a stem-loop RT primer (Applied Biosystems). Quantitative real-time PCR was performed using a TaqMan PCR kit on an Applied Biosystems 7500 Sequence Detection System (Applied Biosystems) with a standard absolute quantification thermal cycling program. The cycle threshold (C_T_) data were determined using fixed threshold settings, and the relative levels of miRNAs in the cells and tissues were normalized to U6. The amount of miRNA relative to the internal U6 control was calculated using the 2^-ΔΔCT^, in which *ΔΔ*C_T_ = (C_T miRNA_ − C_T U6_)_target_ − (C_T miRNA_ − C_T U6_)_control_. To quantify the HNF-3β mRNA, 1 μg of total RNA was reverse-transcribed to cDNA using oligo dT and Thermoscript (TaKaRa), and the real-time PCR was performed using the RT product, SYBER Green Dye (Invitrogen) and specific primers for HNF-3β and β-actin. The relative amount of the HNF-3β mRNA was normalized to β-actin, and the sequences of the primers were as follows: HNF-3β (sense): 5′-CACCACCAGCCCCACAAA-3′; HNF-3β (antisense): 5′-GGGTAGTGCATCACCTGTTCGT-3′; β-actin (sense): 5′-GGCGGCACCACCATGTACCCT-3′; and β-actin (antisense): 5′-AGGGGCCGGACTCG TCATACT-3′.

### The overexpression of miR-141

Synthetic pre-miR-141 and scrambled negative control RNA (pre-miR-control) were purchased from Ambion (Austin, TX, USA). All cells were seeded in 6-well plates or 60-mm dishes. The following day, when the cells were approximately 70% confluent, the cells were transfected with Lipofectamine 2000 (Invitrogen). In each well, equal amounts of pre-miR-141 or pre-miR-control were used. The cells were harvested 24 h after transfection for quantitative RT-PCR and Western blotting.

### Luciferase reporter assay

To test the direct binding of miR-141 to the target gene, HNF-3β, a luciferase reporter assay was performed as previously described [21]. The entire 3′-UTR of human HNF-3β was amplified using PCR with human genomic DNA as a template. The PCR products were inserted into the p-MIR-reporter plasmid (Ambion), and the insertion was confirmed by sequencing. To test the binding specificity, the sequences that interacted with the miR-141 seed sequence were mutated (from AGUGUU to UCACAA), and the mutant HNF-3β 3′-UTR was inserted into an equivalent luciferase reporter. For the luciferase reporter assays, HepG2 cells were cultured in 24-well plates, and cells in each well were transfected with 1 μg of firefly luciferase reporter plasmid, 1 μg of a β-galactosidase (β-gal) expression plasmid (Ambion) and equal amounts (100 pmol) of pre-miR-141 or pre-miR-control using Lipofectamine 2000 (Invitrogen). The β-gal plasmid was used as a transfection control. Twenty-four hours post-transfection, the cells were assayed using a luciferase assay kit (Promega, Madison, WI, USA).

### Plasmid construction and siRNA interference assay

An siRNA sequence targeting human HNF-3β cDNA was designed and synthesized by GenePharma (Shanghai, China); the siRNA sequence was 5′-GAACAUGUCGUCGUACGUG-3′. A scrambled siRNA was included as a negative control. A mammalian expression plasmid encoding the human HNF-3β open reading frame (pReceiver-M02-HNF-3β) was purchased from GeneCopoeia (Germantown, MD, USA), and an empty plasmid served as a negative control. The HNF-3β expression plasmid and HNF-3β siRNA were transfected into HepG2 cells using Lipofectamine 2000 (Invitrogen) according to the manufacturer’s instructions. Total RNA and protein were isolated 24 h post-transfection, and the HNF-3β mRNA and protein expression levels were assessed using quantitative RT-PCR and Western blotting.

### Protein extraction and western blotting

All cells were rinsed with PBS (pH 7.4) and lysed in RIPA Lysis buffer (Beyotime, China) supplemented with a Protease and Phosphatase Inhibitor Cocktail (Thermo Scientific 78440) on ice for 30 min. The tissue samples were frozen solid with liquid nitrogen, ground into a powder and lysed in RIPA Lysis buffer containing the Protease and Phosphatase Inhibitor Cocktail on ice for 30 min. When necessary, sonication was used to facilitate lysis. Cell lysates or tissue homogenates were centrifuged for 10 min (12000 g, 4°C), the supernatant was collected, and the protein concentration was calculated using a Pierce BCA protein assay kit (Thermo Scientific, Rockford, IL, USA). The protein levels were analyzed using Western blotting with the corresponding antibodies and normalized by probing the same blots with a GAPDH antibody. The antibodies were purchased from the following sources: Anti-HNF-3β (Santa Cruz Biotechnology sc-6553, Santa Cruz, CA, USA) and anti-GAPDH (Santa Cruz Biotechnology sc-365062, Santa Cruz, CA, USA). Protein bands were analyzed using the Bandscan ImageJ software.

### Cell proliferation assay

To assess cell proliferation, HepG2 cells were seeded in triplicate in 96-well plates at a density of 5 × 10^3^ cells per well in 100 μL of culture medium. The cell proliferation index was measured using the Cell Counting Kit-8 (CCK-8; Diojindo Laboratories, Kumamoto, Japan) 12, 24, 36 and 48 h after transfection according to the manufacturer’s instructions.

### Cell invasion assay

The invasion ability of HepG2 cells transfected with pre-miR-141 or the HNF-3β overexpression plasmid was tested in a Transwell Boyden Chamber (6.5 mm, Costar, USA). The polycarbonate membranes (8-μm pore size) on the bottom of the upper compartment of the Transwells were coated with 1% human fibronectin (R&D systems 1918-FN, USA). The cells were harvested 24 h after transfection, suspended in FBS-free DMEM culture medium and added to the upper chamber (4 × 10^4^ cells/well). At the same time, 0.5 mL of DMEM with 10% FBS was added to the lower compartment, and the Transwell-containing plates were incubated for 12 h in a 5% CO_2_ atmosphere that was saturated with H_2_O. After incubation, cells that had entered the lower surface of the filter membrane were fixed with 4% paraformaldehyde for 25 min at room temperature, washed 3 times with distilled water and stained with 0.1% crystal violet in 0.1 M borate and 2% ethanol for 15 min at room temperature. Cells remaining on the upper surface of the filter membrane (non-migrant) were scraped off gently with a cotton swab. The lower surfaces (with migrant cells) were imaged using a photomicroscope (5 fields per chamber) (BX51 Olympus, Japan), and the cells were counted blindly.

### Apoptosis assays

The apoptosis of HepG2 cells transfected with pre-miR-141, siRNA or the HNF-3β overexpression plasmid was tested using an Annexin V-FITC/propidium iodide (PI) staining assay. HepG2 cells were cultured in 12-well plates and transfected with pre-miR-141, HNF-3β siRNA or the HNF-3β overexpression plasmid to induce apoptosis. The pre-miR-control, control siRNA and control plasmid served as negative controls. Cells were cultured overnight with serum-containing complete medium and serum-depleted medium, and the attached and floating cells were then harvested. Flow cytometry analysis of apoptotic cells was carried out using an Annexin V-FITC/PI staining kit (BD Biosciences, CA, USA). After washes with cold PBS, the cells were resuspended in binding buffer (100 mM HEPES, pH 7.4; 100 mM NaCl; and 25 mM CaCl_2_) followed by staining with Annexin V-FITC/PI at room temperature in darkness for 15 min. Apoptotic cells were then evaluated by gating PI and Annexin V-positive cells on a fluorescence-activated cell-sorting (FACS) flow cytometer (BD Biosciences, San Jose, CA). All experiments were performed in triplicate.

### Statistical analysis

All of the Western blotting images are representative of at least three independent experiments. Quantitative RT-PCR, the luciferase reporter, the cell proliferation and apoptosis assays were performed in triplicate, and each experiment were repeated several times. The data that are shown are the mean ± SD of at least three independent experiments. The differences were considered statistically significant at p <0.05 using Student’s *t* -test.

## Results

### The upregulation of the HNF-3β protein, but not mRNA, in human HCC tissues

**Figure 1:**
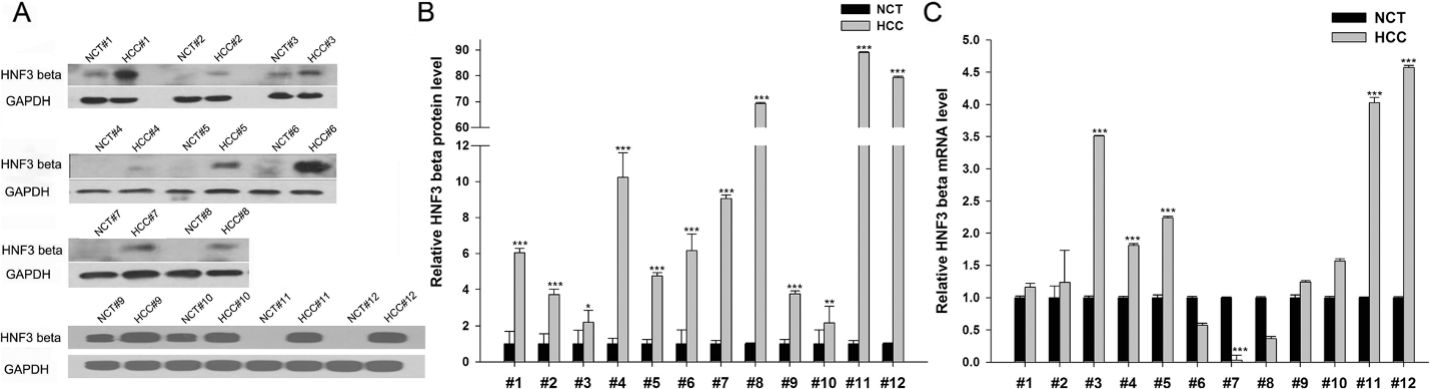
**The expression of HNF-3β in human HCC tissues. (A)** Western blot analysis of the relative HNF-3β protein level in 12 pairs of HCC tissue (HCT) and normal adjacent tissue (NCT) samples. GAPDH was used as a loading panel. **(B)** Quantitative analysis of the data in panel **(A)**. **(C)** Quantitative RT-PCR analysis of the relative HNF-3β mRNA levels in the same 12 pairs of HCT and NCT samples (mean ± S.D.; * p < 0.05; *** p < 0.001).

HNF-3β is in a class of liver-enriched transcription factors that are engaged in the hepatic phenotype. We first determined the expression patterns of the HNF-3β protein in HCC tissues. By measuring the levels of the HNF-3β protein in 12 pairs of HCC tissues using Western blotting, we showed that the expression levels of the HNF-3β protein were significantly higher in tumor tissues than the matched normal tissues (Figure 1A and 1B). Subsequently, we performed quantitative real-time PCR (qRT-PCR) analysis to examine the expression levels of the HNF-3β mRNA in the same tissue samples. We found that the HNF-3β mRNA level appeared to be irregular in tumor specimens than that in normal tissue; however, the overall difference in the HNF-3β mRNA expression level was not statistically significant (Figure 1C). This disparity between the HNF-3β protein and mRNA expression in HCC tissues strongly suggests that a post-transcriptional mechanism is involved in the regulation of HNF-3β.

### Identification of conserved miR-141 target sites within the 3′-UTR of HNF-3β

**Figure 2:**
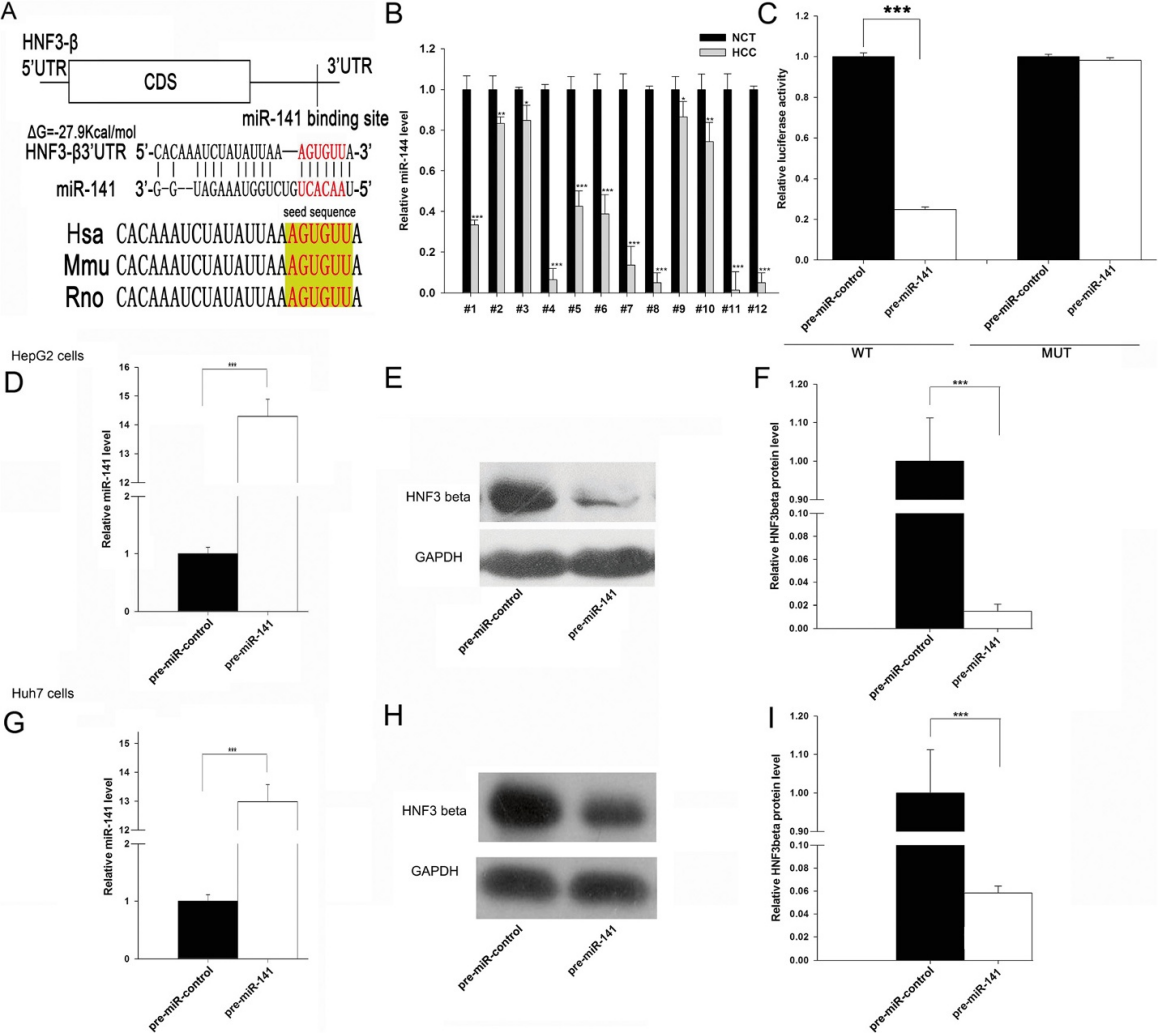
**Prediction and validation of HNF-3β as the target of miR-141. (A)** Schematic description of the hypothesized duplexes formed by the interactions between the HNF-3β 3′-UTR binding site and miR-141. The predicted structure of the base-paired hybrid is diagrammed. Paired bases are indicated by a *black line*, and G:U pairs are indicated by *three dots*. The predicted free energy of the hybrid is indicated. **(B)** Quantitative RT-PCR analysis of the relative miR-141 level in 12 pairs of HCC tissues and noncancerous tissue samples. **(C)** Analysis of luciferase activity. Firefly luciferase reporters containing either the wild-type (WT) or mutant (MUT) form of the human HNF-3β 3′-UTR were cotransfected into HepG2 cells with pre-miR-141 or pre-miR-control. At 24 h post-transfection, the cells were assayed using a luciferase assay kit. Firefly luciferase values were normalized to β-galactoidase activity and plotted as relative luciferase activity. For comparison, the luciferase activity in pre-miR-control-transfected cells was set as 1. **(D)** Quantitative RT-PCR analysis of the relative miR-141 expression level in HepG2 cells transfected with pre-miR-141 or pre-miR-control for 24 h. **(E and F)** Western bolt analysis of the endogenous HNF-3β protein level in HepG2 cells transfected with pre-miR-141 or pre-miR-control for 24 h. **(E)**: representative image; **(F)**: the result of the quantitative analysis. **(G)** Quantitative RT-PCR analysis of the relative miR-141 expression level in Huh7 cells transfected with pre-miR-141 or pre-miR-control for 24 h. **(H and I)** Western bolt analysis of the endogenous HNF-3β protein level in Huh7 cells transfected with pre-miR-141 or pre-miR-control for 24 h. H: representative image; I: the result of the quantitative analysis. (mean ± S.D.; * p < 0.05; *** p < 0.001).

One important mode of post-transcriptional regulation is the repression of mRNA transcripts by miRNAs. miRNAs are, therefore, likely to play a biologically relevant role in regulating HNF-3β expression in HCC. Using three publicly available algorithms (TargetScan, miRanda and PicTar), miR-141 was identified as a candidate miRNA that could target HNF-3β. The predicted interaction between miR-141 and its target site in the HNF-3β 3′-UTR is illustrated in Figure 2A. As shown in this figure, miR-141 has one potential target site in the 3′-UTR of the HNF-3β mRNA sequence. The minimum free energy value of the hybridization is −27.9 kcal/mol, as determined by RNA hybrid analysis, which is well within the range of genuine miRNA-target pairs. Moreover, perfect base-pairing between the seed region (the core sequence that encompasses the first 2–7 bases of the mature miRNA) and the cognate targets was predicted. Furthermore, the miR-141 binding sequences in the HNF-3β 3′-UTR were highly conserved across species.

Next, we investigated whether the levels of miR-141 were inversely correlated with those of HNF-3β in HCC tissues. We measured the expression levels of miR-141 using qRT-PCR in the above-mentioned 12 pairs of tissues. As shown in Figure 2B, miR-141 was significantly lower in human HCC tissues compared with the adjacent normal tissues, consistent with the notion that miRNAs should have expression patterns that are opposite to that of their targets.

### Validation of HNF-3β as a direct target of miR-141

We then determined whether the negative regulatory effect of miR-141 on HNF-3β expression was directly mediated through the binding of miR-141 to the presumed site in the 3′-UTR of the HNF-3β mRNA. The full length HNF-3β 3′-UTR was placed downstream of the firefly luciferase gene in a reporter plasmid. The resulting plasmid was transfected into human HCC cell line HepG2 along with either pre-miR-141 or pre-miR-control. Pre-miR-141 is synthetic RNA oligonucleotides that mimic the miR-141 precursor, which can overexpress miR-141 after being transfected into HepG2 cells. As expected, the luciferase activity was markedly reduced in cells transfected with pre-miR-141 when compared to cells treated with pre-miR-control (Figure 2C). Furthermore, we introduced point mutations into the corresponding complementary sites in the 3′-UTR of HNF-3β to eliminate the predicted miR-141 binding site. Mutation in the complementary seed sites nearly fully rescued the repression of the reporter activity that was caused by the overexpression of pre-miR-141 (Figure 2C).

The correlation between miR-141 and HNF-3β was further examined by evaluating the expression of HNF-3β in the human HCC cell line HepG2 and Huh7 after overexpression of miR-141. HepG2 and Huh7 cells transfected with pre-miR-141 showed a significantly increased expression level of mature miR-141 (Figure 2D and 2G). As anticipated, overexpression of miR-141 significantly reduced the HNF-3β protein levels in HepG2 and Huh7 cells (Figure 2E and 2F; 2H and 2I). Thus, based on computational predictions, their inverse correlation in human cancer tissues and the results of cell transfection assays, HNF-3β was determined to be a miR-141 target.

### The effect of miR-141-mediated downregulation of HNF-3β on cell proliferation, invasion and apoptosis

**Figure 3:**
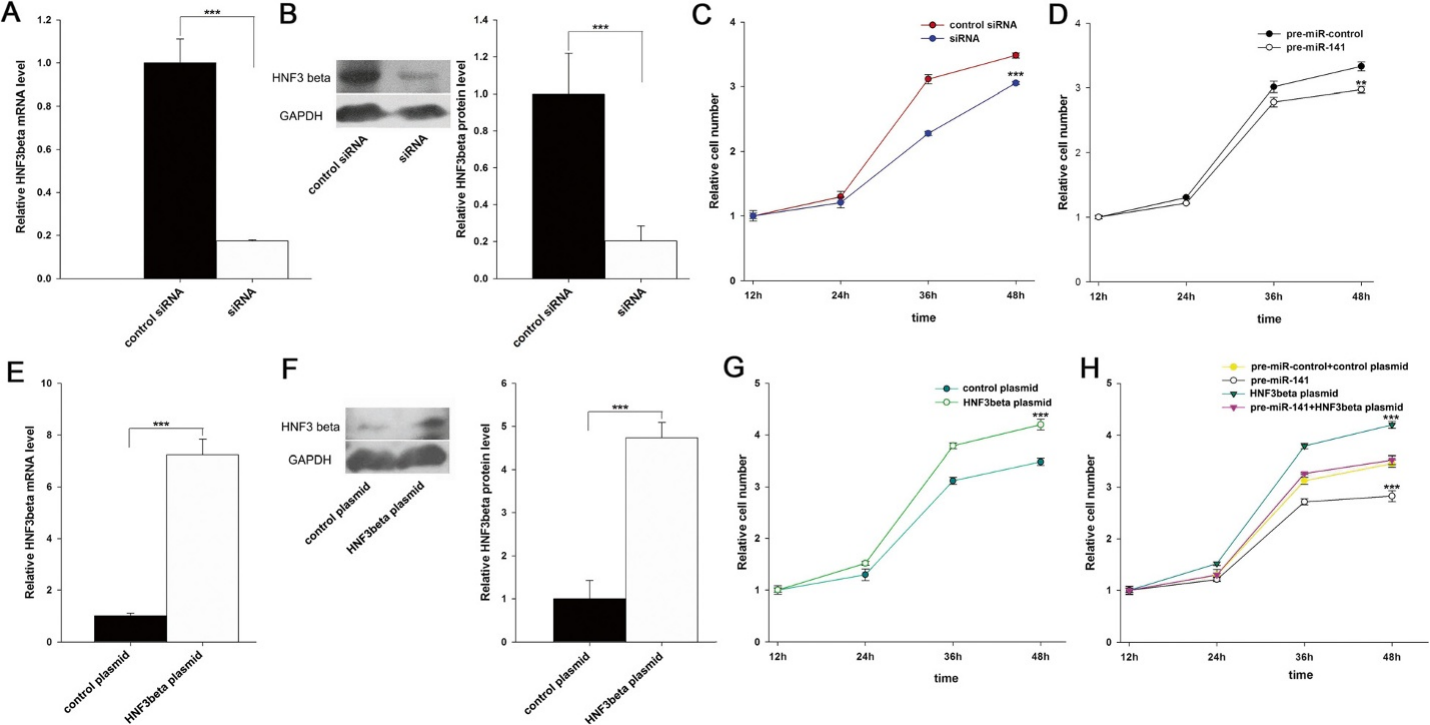
**The effect of miR-141-mediated downregulation of HNF-3β on cell proliferation. (A)** Quantitative RT-PCR analysis of HNF-3β mRNA levels in HepG2 cells when transfected with control or HNF-3β siRNA. **(B)** Western blot analysis of the endogenous HNF-3β protein level in HepG2 cells when transfected with control or HNF-3β siRNA. Left: representative image; right: quantitative analysis. **(C)** Cell proliferation assays were performed 12, 24, 36 and 48 h after transfection of HepG2 cells with scrambled control siRNA or HNF-3β siRNA. **(D)** Cell proliferation assays were performed 12, 24, 36 and 48 h after transfection of HepG2 cells with pre-miR-141 or pre-miR-control. **(E)** Quantitative RT-PCR analysis of the HNF-3β mRNA level in HepG2 cells transfected with the control or HNF-3β plasmid. **(F)** Western blot analysis of the HNF-3β protein level in HepG2 cells transfected with control or HNF-3β plasmid. Left: representative image; right: quantitative analysis. **(G)** Cell proliferation assays were performed 12, 24, 36 and 48 h after transfection of HepG2 cells with control or HNF-3β overexpression plasmid. **(H)** Cell proliferation assays were performed 12, 24, 36 and 48 h after transfection of HepG2 cells with pre-miR-141, HNF-3β plasmid, or pre-miR-141 and the HNF-3β plasmid (mean ± S.D.; ** p < 0.01; *** p < 0.001).

To investigate the cellular phenotypes that are triggered by the miR-141-mediated downregulation of HNF-3β, HepG2 cells were transfected with pre-miR-141, HNF-3β siRNA or the HNF-3β plasmid, and the changes in cell proliferation, invasion and apoptosis were analyzed. Efficient interference of HNF-3β expression could be achieved by transfection of the HNF-3β siRNA (Figure 3A and 3B). We then determined the proliferation rates of HepG2 cells with decreased HNF-3β or overexpressed miR-141 using the Cell Counting Kit-8. Compared with the control siRNA-transfected cells, cells transfected with HNF-3β siRNA proliferated at a significantly lower rate (Figure 3C). Likewise, a significant reduction of the cell proliferation rate was observed in cells transfected with pre-miR-141 (Figure 3D). Subsequently, we investigated whether overexpression of miR-141–resistant HNF-3β (HNF-3β ORF) was sufficient to rescue the suppression of HNF-3β by miR-141 and attenuate the anti-proliferative effect of miR-141 in hepatoma carcinoma cells. Cells transfected with the HNF-3β overexpression plasmid showed increased HNF-3β mRNA and protein levels (Figure 3E and 3F) and proliferation rate (Figure 3G) compared to cells transfected with an empty control plasmid. Consequently, compared to cells transfected with pre-miR-141, cells transfected with pre-miR-141 and the HNF-3β overexpression plasmid exhibited significantly higher proliferation rates (Figure 3H), suggesting that overexpression of HNF-3β rescued the miR-141-mediated downregulation of the proliferation rates of HepG2 cells. Taken together, the results indicate that miR-141 might inhibit cell proliferation by silencing HNF-3β.

**Figure 4:**
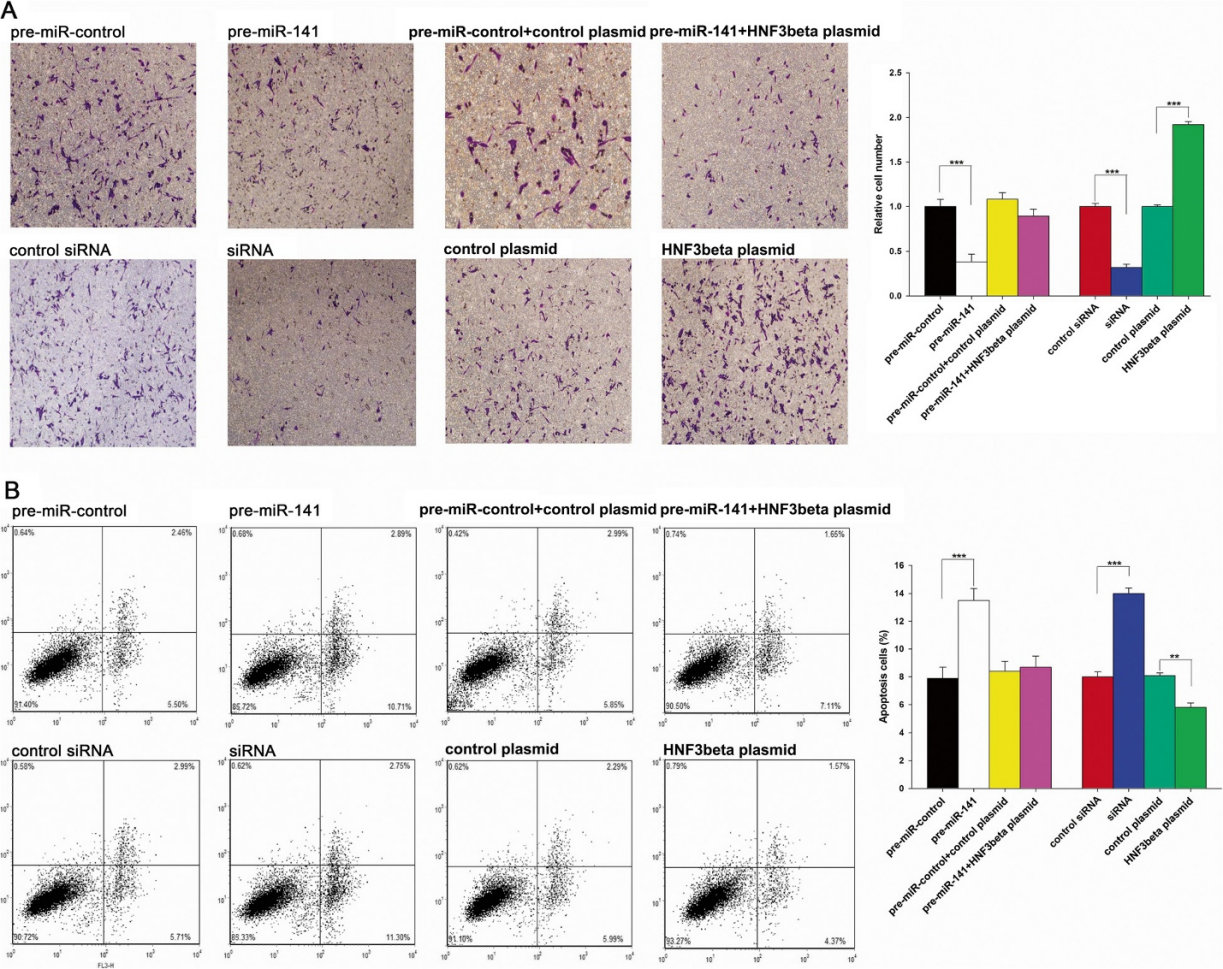
**The effect of miR-141-mediated downregulation of HNF-3β on cell invasion and apoptosis. (A)** Transwell analysis of HepG2 cells treated with equal doses of pre-miR-control, pre-miR-141, scrambled control siRNA, HNF-3β siRNA, scrambled control plasmid, HNF-3β overexpression plasmid or pre-miR-203 plus the HNF-3β overexpression plasmid. The experiment was repeated three times, and the quantitative analysis is shown in the right panel. **(B)** HepG2 cells were transfected with equal doses of pre-miR-control, pre-miR-141, scrambled control siRNA, HNF-3β siRNA, scrambled control plasmid, HNF-3β overexpression plasmid or pre-miR-203 plus the HNF-3β overexpression plasmid. Apoptosis profiles were analyzed by flow cytometry, and the quantitative analysis is shown in the right panel (mean ± S.D.; ** p < 0.01; *** p < 0.001).

Furthermore, we assessed the effect of miR-141 and HNF-3β on the invasion ability of HepG2 cells. The chamber assays showed that the invasion rate of HepG2 cells transfected with pre-miR-141 was significantly decreased when compared to cells transfected with the pre-miR-control (Figure 4A). Additionally, the transfection of the HNF-3β siRNA remarkably reduced the number of HepG2 cells that passed through the Transwell chamber, whereas transfection of the HNF-3β overexpression plasmid increased the invasion rate (Figure 4A). However, when cells were co-transfected with pre-miR-141 and the HNF-3β overexpression plasmid, HNF-3β dramatically attenuated the anti-invasion effect of miR-141 (Figure 4A). These results indicate that miR-141 might inhibit cell invasion by silencing HNF-3β.

We lastly investigated apoptosis in cells with increased miR-141 or silenced HNF-3β expression using flow cytometry analysis. The percentage of apoptotic cells in the pre-miR-141 transfection group was significantly higher when compared to cells transfected with the pre-miR-control (Figure 4B). In addition, the transfection of the HNF-3β siRNA remarkably increased the percentage of apoptotic cells when compared to cells transfected with control siRNA, whereas transfection of the HNF-3β overexpression plasmid decreased apoptosis (Figure 4B). Moreover, compared with cells transfected with pre-miR-141 or the HNF-3β plasmid alone, cells co-transfected with pre-miR-141 and the HNF-3β overexpression plasmid exhibited a normal apoptotic level, suggesting that HNF-3β might reverse the promotive effect of miR-141 on apoptosis. The results indicate that miR-141 might modulate apoptosis by downregulating HNF-3β.

## Discussion

HCC is one of the most highly malignant and lethal cancers of the world [22]. The development and progression of HCC is a complicated process that involves the deregulation of multiple genes that are essential for cell biological processes [23, 24]. The hepatocyte nuclear factor 3 family consists of transcription factors that are enriched in liver and contains three members: HNF-3α, HNF-3β and HNF-3γ [25–27]. The HNF-3 family plays an important role in many biological processes, such as early embryonic development, organ formation and metabolism [28, 29]. As one member of the HNF-3 family, HNF-3β is the first activated gene in the process of embryonic development [30–32]. Reports have found that knockout of HNF-3β in mice can even result in early embryonic death due to the lack of formation of the normal neural notochord [28]. HNF-3β is present in early stages of the pancreas development process, which is essential for pancreas α terminal differentiation and pancreatic β cells secreting insulin [33]. HNF-3β mainly exists in the liver; however, its role in HCC remains to be elucidated. Xu et al. first reported the upregulation of HNF-3β in clinical HCC samples [34]. In this study, we found that HNF-3β protein levels were consistently upregulated in HCC clinical tissues compared with matched, normal adjacent tissues, but HNF-3β mRNA levels varied in random tissues, suggesting that a post-transcriptional mechanism was involved in its regulation. Furthermore, we showed that silencing HNF-3β expression could inhibit cell proliferation and invasion and promote apoptosis in HepG2 cells, while overexpressing HNF-3β had opposite effects on HepG2 cells, indicating its role as an essential oncogene during HCC tumorigenesis.

miRNA is a class of non-coding RNAs that regulates target gene expression at the post-transcriptional level. We used bioinformatic analyses to search for miRNAs that could target HNF-3β and identified miR-141 as a candidate. miR-141 belongs to the miR-200 family and has been reported to be decreased and serve as a tumor suppressor in numerous cancer types [35]. The level of miR-141 showed an inverse correlation with the protein expression of hepatoma-derived growth factor (HDGF) in gastric cancer cells, and overexpression of miR-141 negatively regulated the proliferation and invasion of gastric cancer cells [36]. Yoshino et al. [37] found that miR-141 regulated molecular targets and pathways in human renal cell carcinoma. Zhao et al. [38] reported that miR-141 could inhibit proliferation and invasion by targeting mitogen-activated protein kinase isoform 4 (MAP4K4), which is a member of the mammalian STE20/MAP4K family. Rasheed et al. [39] found that miR-141 was downregulated in prostate cancer cells and had an inverse correlation with the protein expression of G-protein subunit a-13 (GNA13). Forcing overexpression of miR-141 negatively regulated the invasion capability of prostate cancer cells. However, the expression condition and detailed role of miR-141 in HCC are poorly understood, except that miR-141 has been previously reported to suppress the migration and invasion of HCC cells by targeting Tiam1 [40]. In this study, we examined the expression patterns of miR-141 in human HCC tissues and showed that the levels of miR-141 were inversely correlated with those of HNF-3β in HCC tissues. Subsequently, we validated that miR-141 directly recognized the 3′-UTR of the HNF-3β transcript and downregulated HNF-3β expression. We lastly showed that miR-141 inhibited HNF-3β expression, consequently inhibiting cell proliferation and invasion and promoting apoptosis in HepG2 cells. The results delineate a novel regulatory network that employs miR-141 and HNF-3β to fine-tune cell proliferation, invasion and apoptosis in liver cells. We also provided evidence that restoration of HNF-3β expression could reverse miR-141-suppressed cell proliferation and invasion and miR-141-promoted apoptosis, suggesting that the targeting of HNF-3β is a mechanism by which the miR-141 exerts its tumor suppressive function. Therefore, the modulation of HNF-3β by miR-141 may explain, at least in part, why the downregulation of miR-141 during HCC carcinogenesis can promote cancer progression.

Although miR-141 has already been reported to be associated with HCC carcinogenesis, this study reveals a critical role for miR-141 as an inhibitor of cell proliferation and invasion and promoter of apoptosis in HCC cells. More importantly, this study identifies miR-141 as a novel link between the HNF-3β regulatory pathway and HCC and points the important role of miR-141 as a tumor suppressor in HCC through the inhibition of HNF-3β translation. This study also revealed a potential new target for HCC therapy.

## Conclusions

In this study, we found that the expression levels of HNF-3β were significantly higher in HCC clinical tissues compared with matched normal adjacent tissues. In addition, we demonstrated for the first time that HNF-3β is a direct target of miR-141. Finally, we provided evidence that miR-141 could inhibit the proliferation and invasion and promote the apoptosis of HCC cells by silencing HNF-3β. Taken together, our findings provide the first clues regarding the role of miR-141 as a tumor suppressor in cancer cells through the inhibition of HNF-3β translation.

## References

[1] Jemal A, Bray F, Center MM, Ferlay J, Ward E, Forman D (2011). Global cancer statistics. CA Cancer J Clin.

[2] Lin ZZ, Shau WY, Hsu C, Shao YY, Yeh YC, Kuo RN, Hsu CH, Yang JC, Cheng AL, Lai MS (2013). Radiofrequency ablation is superior to ethanol injection in early-stage hepatocellular carcinoma irrespective of tumor size. PLoS ONE.

[3] Lazarevich NL, Cheremnova OA, Varga EV, Ovchinnikov DA, Kudrjavtseva EI, Morozova OV, Fleishman DI, Engelhardt NV, Duncan SA (2004). Progression of HCC in mice is associated with a downregulation in the expression of hepatocyte nuclear factors. Hepatology.

[4] Hashiguchi M, Ueno S, Sakoda M, Iino S, Hiwatashi K, Minami K, Ando K, Mataki Y, Maemura K, Shinchi H, Ishigami S, Natsugoe S (2013). Clinical implication of ZEB-1 and E-cadherin expression in hepatocellular carcinoma (HCC). BMC Cancer.

[5] Clark KL, Halay ED, Lai E, Burley SK (1993). Co-crystal structure of the HNF-3/fork head DNA-recognition motif resembles histone H5. Nature.

[6] Samadani U, Porcella A, Pani L, Johnson PF, Burch JB, Pine R, Costa RH (1995). Cytokine regulation of the liver transcription factor hepatocyte nuclear factor-3 beta is mediated by the C/EBP family and interferon regulatory factor 1. Cell Growth Differ.

[7] Wang K, Brems JJ, Gamelli RL, Holterman AX (2013). Foxa2 may modulate hepatic apoptosis through the cIAP1 pathway. Cell Signal.

[8] Baroukh N, Ravier MA, Loder MK, Hill EV, Bounacer A, Scharfmann R, Rutter GA, Van Obberghen E (2007). MicroRNA-124a regulates Foxa2 expression and intracellular signaling in pancreatic beta-cell lines. J Biol Chem.

[9] Laudadio I, Manfroid I, Achouri Y, Schmidt D, Wilson MD, Cordi S, Thorrez L, Knoops L, Jacquemin P, Schuit F, Pierreux CE, Odom DT, Peers B, Lemaigre FP (2012). A feedback loop between the liver-enriched transcription factor network and miR-122 controls hepatocyte differentiation. Gastroenterology.

[10] Lee RC, Feinbaum RL, Ambros V (1993). The C. elegans heterochronic gene lin-4 encodes small RNAs with antisense complementarity to lin-14. Cell.

[11] Lee RC, Ambros V (2001). An extensive class of small RNAs in Caenorhabditis elegans. Science.

[12] Ambros V, Lee RC, Lavanway A, Williams PT, Jewell D (2003). MicroRNAs and other tiny endogenous RNAs in C. elegans. Curr Biol.

[13] Lytle JR, Yario TA, Steitz JA (2007). Target mRNAs are repressed as efficiently by microRNA-binding sites in the 5′ UTR as in the 3′ UTR. Proc Natl Acad Sci U S A.

[14] Griffiths-Jones S, Grocock RJ, van Dongen S, Bateman A, Enright AJ (2006). miRBase: microRNA sequences, targets and gene nomenclature. Nucleic Acids Res.

[15] Vislovukh A, Vargas TR, Polesskaya A, Groisman I (2014). Role of 3′-untranslated region translational control in cancer development, diagnostics and treatment. World J Biol Chem.

[16] Izzotti A, Pulliero A (2014). The effects of environmental chemical carcinogens on the microRNA machinery. Int J Hyg Environ Health.

[17] Bouyssou JM, Manier S, Huynh D, Issa S, Roccaro AM, Ghobrial IM (2014). Regulation of microRNAs in cancer metastasis. Biochim Biophys Acta.

[18] Fan CG, Wang CM, Tian C, Wang Y, Li L, Sun WS, Li RF, Liu YG (2011). miR-122 inhibits viral replication and cell proliferation in hepatitis B virus-related hepatocellular carcinoma and targets NDRG3. Oncol Rep.

[19] Li L, Tang J, Zhang B, Yang W, Liugao M, Wang R, Tan Y, Fan J, Chang Y, Fu J, Jiang F, Chen C, Yang Y, Gu J, Wu D, Guo L, Cao D, Li H, Cao G, Wu M, Zhang MQ, Chen L, Wang H (2014). Epigenetic modification of MiR-429 promotes liver tumour-initiating cell properties by targeting Rb binding protein 4. Gut.

[20] Zhao N, Wang R, Zhou L, Zhu Y, Gong J, Zhuang SM (2014). MicroRNA-26b suppresses the NF-kappaB signaling and enhances the chemosensitivity of hepatocellular carcinoma cells by targeting TAK1 and TAB3. Mol Cancer.

[21] Yan X, Liang H, Deng T, Zhu K, Zhang S, Wang N, Jiang X, Wang X, Liu R, Zen K, Zhang CY, Ba Y, Chen X (2013). The identification of novel targets of miR-16 and characterization of their biological functions in cancer cells. Mol Cancer.

[22] Wu GG, Li WH, He WG, Jiang N, Zhang GX, Chen W, Yang HF, Liu QL, Huang YN, Zhang L, Zhang T, Zeng XC (2014). Mir-184 post-transcriptionally regulates SOX7 expression and promotes cell proliferation in human hepatocellular carcinoma. PLoS ONE.

[23] Kunter I, Erdal E, Nart D, Yilmaz F, Karademir S, Sagol O, Atabey N (2014). Active form of AKT controls cell proliferation and response to apoptosis in hepatocellular carcinoma. Oncol Rep.

[24] Hong X, Song R, Song H, Zheng T, Wang J, Liang Y, Qi S, Lu Z, Song X, Jiang H, Liu L, Zhang Z (2013). PTEN antagonises Tcl1/hnRNPK-mediated G6PD pre-mRNA splicing which contributes to hepatocarcinogenesis. Gut.

[25] Sasaki H, Hogan BL (1993). Differential expression of multiple fork head related genes during gastrulation and axial pattern formation in the mouse embryo. Development.

[26] Monaghan AP, Kaestner KH, Grau E, Schutz G (1993). Postimplantation expression patterns indicate a role for the mouse forkhead/HNF-3 alpha, beta and gamma genes in determination of the definitive endoderm, chordamesoderm and neuroectoderm. Development.

[27] Ang SL, Wierda A, Wong D, Stevens KA, Cascio S, Rossant J, Zaret KS (1993). The formation and maintenance of the definitive endoderm lineage in the mouse: involvement of HNF3/forkhead proteins. Development.

[28] Weinstein DC, Ruiz i Altaba A, Chen WS, Hoodless P, Prezioso VR, Jessell TM, Darnell JE (1994). The winged-helix transcription factor HNF-3 beta is required for notochord development in the mouse embryo. Cell.

[29] Yamamoto Y, Teratani T, Yamamoto H, Quinn G, Murata S, Ikeda R, Kinoshita K, Matsubara K, Kato T, Ochiya T (2005). Recapitulation of in vivo gene expression during hepatic differentiation from murine embryonic stem cells. Hepatology.

[30] Lai E, Prezioso VR, Tao WF, Chen WS, Darnell JE (1991). Hepatocyte nuclear factor 3 alpha belongs to a gene family in mammals that is homologous to the Drosophila homeotic gene fork head. Genes Dev.

[31] Weigel D, Jurgens G, Kuttner F, Seifert E, Jackle H (1989). The homeotic gene fork head encodes a nuclear protein and is expressed in the terminal regions of the Drosophila embryo. Cell.

[32] Weigel D, Jackle H (1990). The fork head domain: a novel DNA binding motif of eukaryotic transcription factors?. Cell.

[33] Shen W, Scearce LM, Brestelli JE, Sund NJ, Kaestner KH (2001). Foxa3 (hepatocyte nuclear factor 3gamma) is required for the regulation of hepatic GLUT2 expression and the maintenance of glucose homeostasis during a prolonged fast. J Biol Chem.

[34] Xu L, Hui L, Wang S, Gong J, Jin Y, Wang Y, Ji Y, Wu X, Han Z, Hu G (2001). Expression profiling suggested a regulatory role of liver-enriched transcription factors in human hepatocellular carcinoma. Cancer Res.

[35] Xu L, Li Q, Xu D, Wang Q, An Y, Du Q, Zhang J, Zhu Y, Miao Y (2014). hsa-miR-141 downregulates TM4SF1 to inhibit pancreatic cancer cell invasion and migration. Int J Oncol.

[36] Chen B, Huang T, Jiang J, Lv L, Li H, Xia S (2014). miR-141 suppresses proliferation and motility of gastric cancer cells by targeting HDGF. Mol Cell Biochem.

[37] Yoshino H, Enokida H, Itesako T, Tatarano S, Kinoshita T, Fuse M, Kojima S, Nakagawa M, Seki N (2013). Epithelial-mesenchymal transition-related microRNA-200s regulate molecular targets and pathways in renal cell carcinoma. J Hum Genet.

[38] Zhao G, Wang B, Liu Y, Zhang JG, Deng SC, Qin Q, Tian K, Li X, Zhu S, Niu Y, Gong Q, Wang CY (2013). miRNA-141, downregulated in pancreatic cancer, inhibits cell proliferation and invasion by directly targeting MAP4K4. Mol Cancer Ther.

[39] Rasheed SA, Teo CR, Beillard EJ, Voorhoeve PM, Casey PJ (2013). MicroRNA-182 and microRNA-200a control G-protein subunit alpha-13 (GNA13) expression and cell invasion synergistically in prostate cancer cells. J Biol Chem.

[40] Liu Y, Ding Y, Huang J, Wang S, Ni W, Guan J, Li Q, Zhang Y, Ding Y, Chen B, Chen L (2014). MiR-141 suppresses the migration and invasion of HCC cells by targeting Tiam1. PLoS ONE.

[CR41] The pre-publication history for this paper can be accessed here: http://www.biomedcentral.com/1471-2407/14/879/prepub

